# Harnessing delta-like ligand 3: bridging biomarker discovery to next-generation immunotherapies in refractory small cell lung cancer

**DOI:** 10.3389/fimmu.2025.1592291

**Published:** 2025-05-27

**Authors:** Kangkang Ji, Lin Guo, Dianbao Zuo, Mingqian Feng, Xin Chen, Zhenggang Zhao, Jing Tang, Guoping Chen

**Affiliations:** ^1^ Department of Clinical Medical Research, Binhai County People’s Hospital, Binhai Clinical College, Yangzhou University Medical College, Yancheng, Jiangsu, China; ^2^ College of Biomedicine and Health, Huazhong Agricultural University, Wuhan, Hubei, China; ^3^ Department of Respiratory Medicine, Yancheng City No. 6 People’s Hospital, Jiangsu, Yancheng, China; ^4^ Department of Clinical Laboratory, Hubei Rongjun Hospital, Wuhan, Hubei, China; ^5^ Department of Lymphoma, Hubei Cancer Hospital, Tongji Medical College, Huazhong University of Science and Technology, Wuhan, Hubei, China

**Keywords:** DLL3, small cell lung cancer, immunotherapy, biomarker-guided therapy, bispecific T-cell engager

## Abstract

Small cell lung cancer (SCLC), accounting for 10–20% of lung cancers, remains one of the most aggressive neuroendocrine malignancies, with fewer than 7% of patients surviving beyond five years. While the addition of immune checkpoint inhibitors (ICIs) to platinum-based chemotherapy has modestly improved outcomes, long-term benefits are limited to a subset of patients, highlighting the critical need for reliable biomarkers and innovative therapies. Delta-like ligand 3 (DLL3), a Notch signaling regulator overexpressed in 70–80% of SCLC tumors, has emerged as a simultaneous biomarker and therapeutic target. This review aims to synthesize recent advances in DLL3-targeted strategies, bridging biomarker-driven diagnostics to next-generation immunotherapies, while addressing clinical challenges and future directions. The 2024 FDA approval of tarlatamab—a bispecific T-cell engager (BiTE) targeting DLL3 and CD3—marks a pivotal advancement, demonstrating improvement in survival in refractory disease. This review examines three key advances reshaping SCLC management: (1) mechanistic links between DLL3-driven tumorigenesis and PD-L1-mediated immunosuppression, (2) clinical progress in antibody-drug conjugates (ADCs) with next-generation payloads (e.g., FZ-AD005), multispecific BiTEs (e.g., HPN328), and engineered CAR-T/NK cells with enhanced metabolic resilience, and (3) precision strategies combining liquid biopsy for dynamic DLL3 profiling with immuno-PET imaging using [89Zr]Zr-DFO-SC16. Emerging synergies, such as combining DLL3-targeted BiTEs with ICIs to amplify T-cell infiltration or reprogramming CAR-T mitochondrial metabolism, further underscore the potential of multimodal approaches. Together, these developments signal a transformative era in SCLC treatment, where molecular diagnostics and engineered immunotherapies converge to address unmet clinical needs.

## Highlights

- Delta-like ligand 3 (DLL3) serves as a therapeutic target in solid tumors, particularly in neuroendocrine cancers, where its expression is linked to aggressive tumor behavior and poor prognosis.
- Emerging therapies targeting DLL3, such as bispecific T-cell engager (BiTE) and chimeric antigen receptor (CAR) T cells, show promising anti-tumor activity in clinical trials and synergize with immune checkpoint inhibitors.
- DLL3’s molecular heterogeneity across SCLC subtypes highlights its potential as a diagnostic and prognostic biomarker in personalized cancer care.

## 1 Introduction

Small cell lung cancer (SCLC), representing 10-20% of pulmonary malignancies, persists as a formidable clinical challenge with a 5-year survival rate under 7%, underscoring its designation as the most lethal lung cancer subtype (1, 2). Global epidemiology estimates 250,000 annual diagnoses and over 200,000 deaths, driven by aggressive metastatic spread and inherent therapy resistance (3, 4). While the conventional limited-stage (LS) and extensive-stage (ES) stratification informs therapeutic algorithms—with 70% presenting as ES-SCLC (median survival: 10 months)—this system fails to account for molecular heterogeneity (5–7). Current LS-SCLC protocols combining etoposide-cisplatin chemoradiation with prophylactic cranial irradiation achieve transient responses, whereas ES-SCLC first-line treatment incorporates PD-1/PD-L1 inhibitors alongside platinum-based chemotherapy, per IMpower133 and CASPIAN trial frameworks (8–10). Despite these paradigm-shifting approaches, >80% of patients experience relapse within 12 months, with salvage therapies sustaining median progression-free survival below three months (11, 12).

The modest efficacy of immune checkpoint inhibitors (ICIs) in SCLC contrasts sharply with their success in non-small cell lung cancer (NSCLC), a discrepancy attributable to distinct immune evasion mechanisms. SCLC tumors frequently demonstrate major histocompatibility complex (MHC) class I/II downregulation coupled with dynamic NOTCH pathway modifications, collectively fostering an immune-resistant tumor environment (13–16). Although chemoimmunotherapy extends overall survival by 2–4 months in ES-SCLC, these marginal gains highlight the critical need for biomarker- informed therapeutic strategies (17–20).

While traditional antigen discovery focused on genomic aberrations, emerging paradigms emphasize post-translational modifications (PTMs) in generating neoantigenic epitopes. Notably, glycoxidative alterations to histone H2A elicit cancer-specific autoantibody responses, establishing PTMs as key immunogenic drivers (21). This mechanistic framework extends to surface oncoproteins like Delta-like ligand 3 (DLL3), which exhibits tumor-selective overexpression in 70–80% of SCLC cases versus negligible normal tissue expression—establishing its dual role as a biomarker and therapeutic target with intrinsic immunogenic potential (22–25).

Functioning as a non-canonical Notch modulator, DLL3 demonstrates context-dependent oncobiology in SCLC, serving paradoxical roles as both tumorigenic driver and metastasis suppressor (10, 26–28). Its functional duality, combined with microenvironmental PD-L1 co-expression patterns, reinforces DLL3’s utility as a theranostic target (28, 29). Early therapeutic efforts prioritized antibody-drug conjugates (ADCs) like rovalpituzumab tesirine (Rova-T), which showed initial antitumor activity but faced discontinuation due to payload-associated toxicities (30–33). Subsequent innovations yielded tarlatamab, a DLL3-guided T-cell activator approved by the FDA in 2024 for platinum-refractory ES-SCLC, leveraging its optimized pharmacokinetics and favorable safety profile (34–37). Parallel advancements include trispecific T-cell engagers (HPN328) and CAR-T/NK platforms demonstrating complete tumor regression in disseminated SCLC models (38–41). Despite these breakthroughs, predictive biomarkers for DLL3-targeted therapies remain undefined, necessitating molecular profiling approaches to refine patient selection.

To ensure a comprehensive synthesis of recent advancements, this review systematically evaluated peer-reviewed articles (PubMed, Web of Science; 2010–2025), registered clinical trials (ClinicalTrials.gov), and preclinical studies, prioritizing high-impact journals and FDA-approved therapies (e.g., tarlatamab). Search terms included ‘DLL3,’ ‘SCLC,’ ‘BiTE,’ and ‘CAR-T,’ with exclusion of non-English or non-peer-reviewed sources. This review synthesizes the evolving role of DLL3 in SCLC therapeutics, critically evaluating current clinical strategies and future directions.

## 2 Recent advancements in DLL3 as a diagnostic biomarker

The diagnostic evaluation of SCLC faces inherent challenges due to the difficulty of obtaining tumor tissue biopsies, which are further compounded by the aggressive biology of this malignancy (42, 43). Liquid biopsy has emerged as a critical alternative, particularly given SCLC’s rapid progression (median tumor doubling time <90 days) and frequent presentation with metastatic disease (44–46). Circulating tumor cells (CTCs) demonstrate particular utility in this context, enabling serial molecular profiling that aligns with the dynamic evolution of SCLC under therapeutic pressure (47). While CTCs may exhibit phenotypic divergence from primary tumor cells (48, 49), their capacity to reflect real-time tumor biology positions liquid biopsy as an indispensable tool for monitoring DLL3 expression dynamics during treatment (50).

DLL3’s diagnostic potential is underscored by its overexpression in >75% of SCLC tumors, with expression patterns conserved across limited- and extensive-stage disease (29, 33, 51, 52). The largest systematic review to date (Bylsma et al., 2023) reported DLL3 positivity in 80–93.5% of SCLC tumors using a threshold of ≥1% tumor cells and 58.3–91.1% with ≥25% expression. High expression (≥50% or ≥75%) was observed in 45.8–79.5% and 47.3–75.6% of cases, respectively. Notably, no consistent associations were found between DLL3 levels and patient demographics (age, sex, smoking) or survival outcomes, underscoring the need for standardized biomarker validation in therapeutic targeting (53). This biomarker prevalence exceeds traditional neuroendocrine markers like chromogranin A, offering improved diagnostic sensitivity (54). Clinical correlates reveal that elevated DLL3 expression associates with inferior outcomes in ES-SCLC and predicts enhanced response to DLL3-targeted therapies—patients with >75% DLL3+ tumor cells demonstrated a 38% objective response rate to Rova-T versus 11% in low expressers (29, 55).

However, the biomarker’s pathophysiological duality complicates interpretation: while promoting metastasis via Snail-mediated EMT, DLL3 simultaneously correlates with immunosuppressive tumor microenvironment (TME) features including decreased CD8+ T-cell infiltration and increased PD-L1 expression (27, 56). In addition, emerging clinical evidence challenges the exclusivity of DLL3 as a predictive biomarker. The phase II DeLLphi-301 trial demonstrated that tarlatamab, a DLL3/CD3 BiTE, achieved comparable response rates (40% vs. 32%) and progression-free survival (4.9 vs. 3.9 months) across SCLC patients irrespective of DLL3 expression thresholds, suggesting the involvement of DLL3-independent immune activation mechanisms (57). This paradox highlights the need to reconcile DLL3’s diagnostic prominence with its limited predictive power in BiTE therapies, necessitating multimodal biomarker strategies to optimize patient selection.

Technological innovations are resolving key barriers to clinical implementation. Sharma et al. pioneered DLL3-targeted PET imaging using 89Zr-labeled SC16.56 antibodies, achieving 92% sensitivity in detecting DLL3+ metastases while overcoming the sampling bias of biopsy-based approaches (58). Subsequent validation in the phase I/II ZENITH trial (NCT04199741) demonstrated [89Zr]Zr-DFO-SC16.56’s ability to discriminate DLL3 expression levels across metastatic sites, with tumor-to-background ratios >5.0 predicting Rova-T response (59). Standardization efforts now focus on harmonizing detection platforms—comparative analyses reveal immunohistochemistry (IHC) H-scores show strong correlation with RNAscope quantification (r=0.81) and NanoString digital spatial profiling (r=0.76), though inter-laboratory variability remains a concern (60–62).

The integration of CTC-based DLL3 monitoring with radiographic biomarkers presents a transformative opportunity for adaptive treatment strategies. Longitudinal studies reveal CTC DLL3 expression fluctuates during therapy, with >50% reductions predicting radiographic response 8 weeks earlier than conventional CT metrics (63). When combined with immuno-PET, this multi-modal approach achieves 94% accuracy in identifying DLL3-avid clones resistant to PD-1 inhibitors, enabling timely escalation to BiTE or CAR-T therapies (58, 59, 64, 65). While challenges persist in defining universal expression thresholds, contemporary advances position DLL3 as the cornerstone of next-generation SCLC diagnostic frameworks (**Table 1**).

**Table 1:** Comparative analysis of DLL3 biomarker detection methods.

Method	Sensitivity	Specificity	Clinical Utility	Limitations	Reference
IHC	High	High	Standard for tissue-based DLL3 quantification	Inter-lab variability	(52)
Liquid Biopsy (CTC)	Moderate	High	Real-time monitoring; correlates with treatment response	Limited in low tumor burden	(49)
Immuno-PET ([89Zr]Zr-DFO-SC16)	High	High	Non-invasive; detects heterogeneous DLL3 expression	Requires specialized imaging	(58)
RNAscope	High	High	High-resolution spatial profiling	Tissue fixation artifacts	(61)

## 3 Cutting-edge developments and pioneering achievements in DLL3-targeted therapies for SCLC

**Figure 1:**
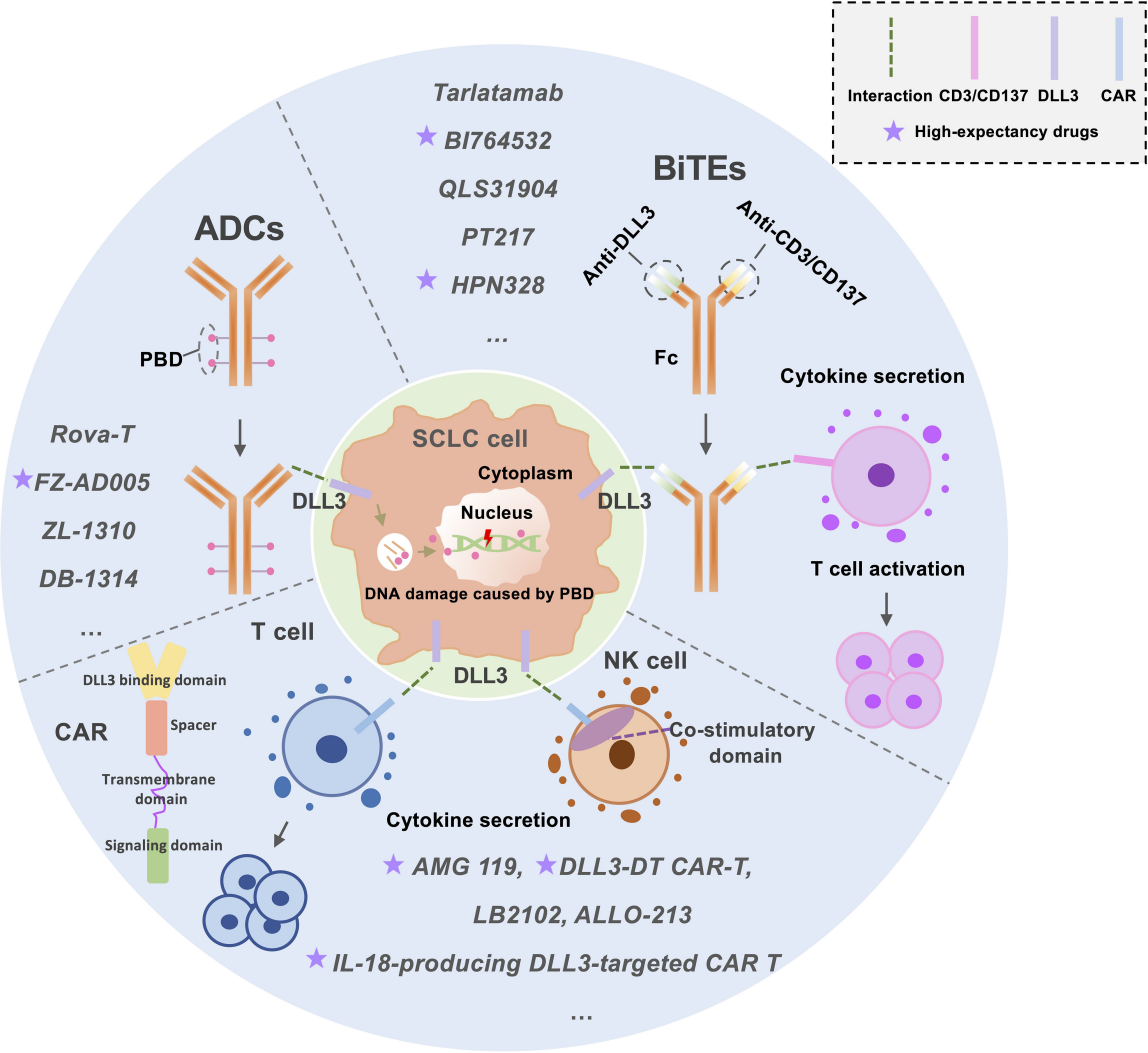
An Overview of the Current Status of DLL3-Targeted Drug Development for SCLC Treatment. This figure illustrates the current landscape of DLL3-targeted therapies for small-cell lung cancer (SCLC) treatment, focusing on three key approaches: Antibody-Drug Conjugates (ADCs): ADCs deliver cytotoxic agents, such as pyrrolobenzodiazepine (PBD), to tumor cells. These agents induce DNA damage, targeting cancer cells while sparing healthy tissue. Bispecific T-Cell Engagers (BiTEs): BiTEs function by simultaneously binding to DLL3 on tumor cells and CD3/CD137 on T-cells. This dual targeting activates T-cells, enhancing their anti-tumor activity. Chimeric Antigen Receptor (CAR) T/NK-Cells: CAR T/NK-cells are engineered to express receptors that recognize DLL3 on tumor surfaces. Upon binding, these cells activate and replicate, leading to robust anti-tumor activity. This figure highlights the diverse mechanisms employed in DLL3-targeted therapies, offering insights into their potential for effective SCLC treatment. ADCs, Antibody-drug conjugates; BiTEs, Bispecific T-cell engagers; CAR T-cell, Chimeric antigen receptor T-cell; PBD, Pyrrolobenzodiazepine.

DLL3, a Notch inhibitory ligand selectively overexpressed on the surface of small cell lung cancer (SCLC) cells, has emerged as a promising therapeutic target. Current strategies include ADCs and immunotherapies such as BiTEs and chimeric antigen receptor T cells (CAR-Ts), which redirect immune cytotoxicity toward DLL3-expressing tumors (66, 67) (**Figure 1**). This section highlights recent advancements in DLL3-targeted therapies, focusing on preclinical and clinical progress.

### 3.1 Antibody-drug conjugates

#### 3.1.1 Rova-T

Rova-T, the first DLL3-targeting ADC developed for extensive-stage SCLC (ES-SCLC), combines a humanized monoclonal antibody with a DNA cross-linker payload via a protease-cleavable linker (10, 26, 68, 69). Its dual mechanism targets cancer stem cells via DLL3 binding and eliminates bulk tumor cells through cytotoxic payload delivery (68). Initial phase I trials (NCT01901653) demonstrated promising antitumor activity and tolerability in pretreated SCLC patients (33). However, the subsequent phase II TRINITY trial (NCT02674568) revealed significant toxicity, with 63% of participants experiencing grade 3–5 adverse events (e.g., pleural effusions, photosensitivity) (29). Two phase III trials (MERU: NCT03033511; TAHOE: NCT03061812) further showed no survival benefit compared to standard therapies, coupled with severe toxicities such as edema and thrombocytopenia, ultimately halting its clinical development (30, 57, 70).

#### 3.1.2 FZ-AD005

To address Rova-T’s limitations, FZ-AD005 was designed with a topoisomerase I inhibitor (DXd) payload, Fc-silencing technology to reduce off-target effects, and an optimized drug-to-antibody ratio (DAR=8.0) for enhanced tumor delivery (71). Preclinical studies demonstrated potent DLL3-specific antitumor activity in both cell line-derived and patient-derived xenograft models, with improved safety profiles attributed to reduced Fc-mediated toxicity (71). A first-in-human clinical trial, approved in April 2024, is underway to evaluate its therapeutic potential.

#### 3.1.3 ZL-1310 and DB-1314

ZL-1310, a novel ADC employing camptothecin derivatives, exhibits high DLL3 specificity without cross-reactivity to other Delta ligands. Preclinical studies revealed dose-dependent tumor suppression and favorable *in vivo* tolerability (72). A phase Ia/Ib multicenter trial (NCT05557122) is currently assessing its pharmacokinetics and safety in SCLC patients (73). Similarly, DB-1314 incorporates a humanized anti-DLL3 antibody conjugated to a topoisomerase I inhibitor (P1021), inducing robust DNA damage and sustained antitumor effects in preclinical models (74). Its enhanced cytotoxic potency, compared to Rova-T, positions it as a potential breakthrough in overcoming prior therapeutic shortcomings (74). A key innovation of DB-1314 compared to Rova-T is the use of P1021 as a cytotoxic warhead to drive the cytotoxic activity of DB-1314 by inducing more potent DNA damage, which may enable DB-1314 to achieve success not seen with Rova-T.

This evolving landscape underscores the need for optimized ADC designs to balance efficacy with manageable toxicity, paving the way for next-generation DLL3-targeted therapies in SCLC.

### 3.2 BiTE therapies

#### 3.2.1 Tarlatamab (AMG757)

BiTEs are composed of two single-chain fragment variables (scFvs)—one targeting tumor antigens (e.g., DLL3) and another engaging T-cell CD3—along with an Fc region to prolong serum half-life (57). Tarlatamab (AMG757), a DLL3-targeting BiTE engineered by Giffin’s team, demonstrates enhanced pharmacokinetics and low picomolar potency against SCLC cells *in vitro*, coupled with robust tumor growth inhibition in preclinical models (75). As the first-in-class DLL3 BiTE, tarlatamab recruits T cells to DLL3-expressing tumors, triggering MHC-I-independent activation, granzyme/perforin release, and tumor lysis (26, 76). Clinical trials in pretreated SCLC patients revealed manageable toxicity profiles, though neutropenia emerged as an unexpected risk (76). A phase II study (10 mg biweekly) reported durable objective responses, survival benefits, and no new safety signals, supporting its antitumor efficacy (77). To optimize outcomes, ongoing trials are evaluating tarlatamab combined with PD-1/PD-L1 inhibitors (NCT03319940, NCT04885998, NCT05740566, and NCT05361395) (40). Notably, tarlatamab recently gained U.S. approval for extensive-stage SCLC progressing during or after chemotherapy, a landmark achievement in the field (34, 35).

#### 3.2.2 BI764532, QLS31904, and PT217

Building on the success of AMG757, several novel BiTE constructs targeting DLL3 have advanced in SCLC research. BI764532, an IgG-like DLL3/CD3 T-cell engager (TCE), demonstrates potent antitumor activity in DLL3+ cell lines and xenograft models, inducing T-cell infiltration and complete tumor regression in humanized mouse models (37, 78). A phase I dose-escalation trial (NCT04429087) is currently evaluating its safety and preliminary efficacy in locally advanced or metastatic DLL3+ SCLC (79). QLS31904, another DLL3/CD3 BiTE developed by Yang et al., achieves complete tumor regression in xenograft models at low doses (20 µg/kg) via CD3-mediated T-cell priming and activation (80). Concurrently, PT217—a DLL3/CD47 bispecific antibody with Fc effector function—blocks CD47-mediated immune evasion while enhancing macrophage phagocytosis (ADCP) and NK cell cytotoxicity (ADCC) through Fc engagement, showing preclinical efficacy with minimal hematotoxicity (81). The initiation of a PT217 phase I trial and its FDA orphan drug designation highlight its therapeutic potential despite remaining developmental challenges (10).

### 3.3 Trispecific T-cell engagers

#### 3.3.1 HPN328

HPN328, a trispecific T-cell activation construct (TriTAC), integrates three functional domains: a DLL3-targeting module for tumor binding, an albumin-binding domain to prolong serum half-life, and a CD3-binding domain for T-cell engagement (38). This innovative T-cell engager (TCE) platform combines the extended pharmacokinetic profile of traditional BiTEs with enhanced tumor penetration due to its compact molecular size and structural stability (82, 83). Preclinical studies confirmed HPN328’s potent, dose-dependent cytotoxicity against DLL3-expressing SCLC cells *in vitro*, coupled with robust T-cell activation and cytokine release (38). A phase 1/2 trial (NCT04471727) evaluating HPN328 monotherapy in advanced DLL3+ malignancies, including ES-SCLC, has reported interim results: 40% of SCLC patients achieved target lesion reduction, with no grade ≥3 adverse events observed in responders (>30% tumor shrinkage), while 20% exhibited disease stabilization (84). These findings underscore its potential as a next-generation TCE for solid tumors.

#### 3.3.2 DLL3/CD3/CD137 trispecific antibody

While BiTEs show promise in immune checkpoint inhibitor (ICI)-resistant cancers, their efficacy remains inconsistent in SCLC (85, 86). To address this limitation, Mikami et al. pioneered a trispecific antibody design by engineering a CD3/CD137 (4-1BB) bispecific Fab scaffold. This foundation enables competitive binding to CD3 and CD137, preventing off-target DLL3 cross-linking while enhancing tumor-specific T-cell activation. Preclinical studies demonstrated superior tumor control and increased intratumoral T-cell infiltration compared to conventional DLL3 BiTEs (87). Building on these results, a phase I trial (NCT05619744) is evaluating RG6524 (RO7616789), a trispecific DLL3-targeted antibody, in SCLC patients.

### 3.4 DLL3-targeted CAR T-cell therapy

CAR T-cell therapy involves engineering patient-derived T cells to express receptors targeting tumor-specific antigens like DLL3 (88). While CAR T-cell therapies have achieved success in refractory B-cell malignancies, their application in solid tumors such as SCLC remains limited due to challenges like T-cell exhaustion and poor tumor infiltration (89–91). Compared to BiTE therapies, CAR T cells offer advantages including sustained *in vivo* expansion, prolonged activity, and potential for combination with anti-aging strategies (92). Regional delivery approaches have shown promise in enhancing CAR T-cell proliferation, infiltration, and systemic immunity in solid tumors (92, 93). DLL3’s consistent expression in newly diagnosed, recurrent, and treatment-resistant SCLC positions it as an ideal target for CAR T-cell immunotherapy (94–96). Preclinical studies by Zhang et al. demonstrated that allogeneic DLL3 CAR T cells exhibit potent specificity and cytotoxicity against SCLC cell lines, even those with extremely low DLL3 expression (≤1000 molecules/cell), mirroring levels observed in primary tumors (41). *In vivo*, DLL3 CAR T-cell infusion induced complete tumor regression in systemic and subcutaneous SCLC models (41), with efficacy further validated in orthotopic and metastatic settings, suggesting clinical potential for ES-SCLC (40).

The second-generation DLL3-targeted CAR T-cell therapy AMG119, incorporating a 4-1BB (CD137) costimulatory domain, demonstrated durable tumor-killing effects in preclinical SCLC xenograft models (97, 98). An early-phase trial in relapsed/refractory SCLC patients post-platinum therapy reported a favorable safety profile and preliminary antitumor activity, though a combination study with tarlatamab (NCT03392064) was halted due to low enrollment (10, 66, 97). To overcome CAR T-cell limitations, IL-18-engineered DLL3-targeted CAR T cells were developed, showing enhanced activation, reduced exhaustion, and synergistic efficacy with anti-PD-1 therapy in preclinical models (99). Additionally, DLL3-TREM1/DAP12 CAR-T (DLL3-DT CAR-T) demonstrated improved memory phenotypes and antigen-presenting cell activation *in vitro* (9). Emerging candidates under investigation include LB2102 (NCT05680922) and ALLO-213 (57, 100). These advances underscore the evolving potential of DLL3-targeted CAR T-cell therapies for SCLC (**Table 2**).

**Table 2:** Key clinical trials of DLL3-Targeted therapies in SCLC.

Therapy	Type	Clinical Phase	Key Findings	Reference
Rova-T	ADC	Phase II (TRINITY)	ORR: 14.3% in high DLL3 expressers; severe toxicity	(29)
Tarlatamab (AMG757)	BiTE (DLL3/CD3)	Phase I/II	Durable responses in refractory SCLC; manageable safety profile	(77)
HPN328	TriTAC	Phase I/II	40% lesion reduction in SCLC; no grade ≥3 adverse events in responders	(84)
ZL-1310	ADC	Phase Ia/Ib	Preclinical tumor suppression; ongoing evaluation of pharmacokinetics/safety	(73)

ORR, objective response rate.

### 3.5 DLL3-targeted CAR NK-cell therapy

CAR NK-cell therapy represents a promising alternative to CAR T-cell approaches in SCLC, offering distinct advantages such as allogeneic compatibility (eliminating the need for MHC matching) and dual tumor-killing mechanisms—both CAR-dependent targeting and innate CAR-independent cytotoxicity—while minimizing risks of cytokine release syndrome and neurotoxicity (101). NK-92 cells, a widely studied CAR NK platform, are further enhanced by the NKG2D transmembrane domain and the 2B4-CD3 co-stimulatory molecule, amplifying their cytotoxic potential (102–104). These features have propelled CAR NK-92 cells into clinical exploration for solid tumors. Preclinical studies of DLL3-targeted CAR NK-92 cells demonstrated potent antitumor activity and robust infiltration in SCLC subcutaneous xenograft models (39). Building on these findings, a phase I trial (NCT05507593) evaluating DLL3-CAR NK-cell therapy in ES-SCLC patients has been initiated, marking a critical milestone in translating this off-the-shelf approach to clinical practice.

### 3.6 Other promising therapies targeting DLL3

Targeted radiotherapy using the radioimmunoconjugate[177Lu]Lu-DTPA-CHX-A-SC16—a lutetium-177-labeled DLL3 monoclonal antibody—demonstrates precise binding to DLL3 on SCLC cells, enabling localized radiation delivery while sparing healthy tissues, a breakthrough in precision oncology (105). Another innovative approach, near-infrared photoimmunotherapy (NIR-PIT), combines antibody-mediated targeting with photodynamic ablation. This strategy employs conjugates such as “rova-IR700,” formed by linking the anti-DLL3 antibody rovalpituzumab to the photosensitizer IR700. Upon near-infrared light exposure, rova-IR700 selectively induces rapid cancer cell death in DLL3-overexpressing SCLC models while preserving normal cells, highlighting its therapeutic specificity (106, 107).

### 3.7 Molecular mechanisms of DLL3 signaling and therapeutic vulnerability

DLL3, a non-canonical inhibitor of the Notch signaling pathway, exhibits unique molecular mechanisms that underpin its dual role in small cell lung cancer (SCLC) progression and therapeutic targeting. Structurally distinct from other Delta-like family members, DLL3 lacks the canonical DSL (Delta-Serrate-Lag2) domain required for Notch receptor activation, instead forming intracellular interactions with Notch ligands and receptors to inhibit signaling via ligand cis-inhibition and receptor retention in the Golgi apparatus (26, 95). This Notch-suppressive activity drives the maintenance of neuroendocrine differentiation and stem-like properties in SCLC, as evidenced by ASCL1-dependent transcriptional upregulation of DLL3 in tumor-initiating cells (26, 27, 108). Preclinical studies demonstrate that DLL3 overexpression promotes epithelial-mesenchymal transition (EMT) through Snail-mediated repression of E-cadherin, enhancing metastatic potential while paradoxically correlating with reduced CD8+ T-cell infiltration and elevated PD-L1 expression in the TME (27, 56).

Therapeutically, DLL3’s tumor-specific membrane localization and minimal normal tissue expression render it an ideal target for precision therapies. However, its role in immune evasion complicates therapeutic outcomes. Mechanistic studies reveal that DLL3-high SCLC tumors exhibit STAT3-driven PD-L1 upregulation, creating an immunosuppressive niche resistant to immune checkpoint inhibitors (ICIs) (56). This duality is exploited by BiTEs like tarlatamab, which redirect T cells to DLL3+ tumors while bypassing MHC-I downregulation—a common immune evasion mechanism in SCLC (36, 77). Preclinical models further demonstrate that DLL3-targeted BiTEs induce T-cell activation and cytokine secretion, synergizing with PD-1/PD-L1 blockade to overcome adaptive immune resistance (85, 109).

DLL3’s therapeutic vulnerability is also influenced by molecular heterogeneity across SCLC subtypes. ASCL1- and NEUROD1-driven tumors exhibit robust DLL3 expression, whereas POU2F3-defined subtypes show reduced levels, correlating with differential responses to DLL3-directed therapies (110, 111). This heterogeneity underscores the importance of dynamic biomarker monitoring, as chemotherapy and targeted therapies can epigenetically suppress DLL3 via DNA methylation, reducing target availability (26, 32). Liquid biopsy studies reveal that circulating tumor cell (CTC) DLL3 expression fluctuates during treatment, with ≥50% reductions predictive of early radiographic response, enabling real-time therapeutic adaptation (49, 63).

Emerging strategies to address DLL3-driven resistance focus on combinatorial approaches and cellular engineering. For instance, IL-18-secreting DLL3 CAR-T cells counteract TME immunosuppression by enhancing T-cell persistence and reversing exhaustion, achieving complete tumor regression in preclinical models (99). Similarly, trispecific antibodies targeting DLL3, CD3, and CD137 amplify T-cell co-stimulation, demonstrating superior tumor control compared to conventional BiTEs *in vivo* (87). These advances highlight the convergence of molecular insights and engineered immunotherapies in exploiting DLL3’s signaling paradox for clinical benefit.

## 4 Role of DLL3 in multiple tumors

Emerging evidence reveals DLL3 as a pan-cancer oncoprotein with context-dependent therapeutic implications across diverse malignancies. Its expression patterns demonstrate remarkable tissue specificity, ranging from ubiquitous surface presentation in neuroendocrine tumors to epigenetic silencing in certain carcinomas. While DLL3-targeted therapies show particular promise in tumors with high antigen homogeneity like SCLC, their clinical application in other cancers requires careful consideration of tumor microenvironment complexities, blood-brain barrier limitations, and compensatory signaling pathways. The following sections systematically analyze DLL3’s multifaceted roles in various neoplasms, highlighting both diagnostic/prognostic utilities and therapeutic vulnerabilities.

### 4.1 Neuroendocrine carcinomas

DLL3 exhibits broad expression across neuroendocrine carcinomas (NECs), encompassing high-grade pulmonary, mammary, pancreatic, prostatic, and intestinal NECs, as well as low-grade gliomas and neuroblastomas, establishing its viability as a surface antigen for targeted therapies (51, 99). In gastroenteropancreatic neuroendocrine neoplasms (GEP-NENs)—a rare, heterogeneous entity with variable clinical trajectories—DLL3 serves dual diagnostic roles. Early investigations localized its expression to neuroendocrine cells, suggesting involvement in gastrointestinal oncogenesis (112), while subsequent studies validated its utility in histological differentiation of poorly differentiated NEC subtypes, including GEP-NENs (113, 114).

### 4.2 NEPC and CRPC

DLL3 overexpression characterizes neuroendocrine prostate cancer (NEPC), an aggressive variant with constrained therapeutic options (115). Korsen et al. pioneered translational applications through the development of ¹^77^Lu-DTPA-SC16, a lutetium-177-conjugated DLL3-targeting antibody demonstrating clinical efficacy (116), complemented by ^89^Zr-DFO-SC16 for PET-based detection of DLL3-positive lesions (65). In medullary thyroid carcinoma (MTC), DLL3 overexpression (≥50% tumor positivity) associates with diminished overall and disease-free survival, particularly in cases exhibiting exfoliated cells, reinforcing its prognostic relevance in aggressive disease (117). Comparative analysis reveals unique challenges in NEPC-targeted therapy versus SCLC, including reduced antigen homogeneity (≤50% positivity in 60% of cases) and competition with established targets like PSMA (65, 118). Although preclinical models show efficacy of [¹^77^Lu]Lu-DTPA-SC16 in NEPC (116), clinical translation necessitates strategies to overcome tumor heterogeneity and microenvironment-mediated resistance.

DLL3 similarly features prominently in castration-resistant prostate cancer (CRPC), where expression correlates with epigenetic markers such as H3K27me3, suggesting mechanistic involvement in disease progression (25, 119). Multi-omics analyses across pulmonary carcinoids, large cell neuroendocrine carcinomas (LCNEC), and SCLC further substantiate DLL3’s therapeutic candidacy (113). Its marked overexpression in bladder small-cell carcinomas and LCNEC underscores broad applicability in rare, aggressive malignancies (120, 121).

### 4.3 Gliomas

Despite uniform DLL3 expression in IDH-mutant gliomas, therapeutic exploitation remains constrained by blood-brain barrier impermeability and NOTCH pathway redundancy. Rova-T’s limited efficacy in glioblastoma—attributed to suboptimal tumor penetration and compensatory signaling—exemplifies these challenges (122, 123).

### 4.4 Breast and hepatocellular carcinomas

Beyond neuroendocrine malignancies, elevated DLL3 expression portends adverse prognosis and enhanced immune infiltration in invasive breast cancer (124). Hepatocellular carcinoma (HCC) presents a paradoxical scenario: epigenetic silencing via DNA methylation and histone acetylation facilitates immune evasion, while forced overexpression induces apoptosis (125). Metastatic analyses further associate DLL3 expression in treatment-emergent small cell/NEPCs with survival reduction (118). This tissue-specific regulation, contrasting with SCLC’s constitutive expression, emphasizes context-dependent targetability (125).

### 4.5 Therapeutic context-dependency

Collectively, these findings emphasize the necessity for tumor-specific therapeutic designs. While SCLC’s high DLL3 homogeneity enables potent responses to T-cell engagers like tarlatamab (77), gliomas and CRPC may require combinatorial approaches with epigenetic modulators to circumvent resistance mechanisms (118, 122).

## 5 Challenges and controversies in DLL3-targeted therapy

Despite advancements, DLL3-targeted therapies face interrelated scientific and clinical hurdles. The functional duality of DLL3—suppressing metastasis while fostering immune evasion—creates a therapeutic paradox requiring context-specific strategies (27, 56). Clinical setbacks, exemplified by Rova-T’s toxicity and BiTE-associated CRS, underscore the need for optimized payloads and dosing regimens (29, 77). Similarly, CAR-T/NK therapies, though promising, grapple with TME-driven resistance and manufacturing scalability (40, 99). Future success hinges on three pillars: (1) biomarker-guided combinatorial regimens (e.g., BiTEs + ICIs) to counteract compensatory immune checkpoints; (2) engineered cellular therapies with metabolic resilience; and (3) real-time monitoring via liquid biopsy and immuno-PET to track DLL3 dynamics. These priorities, rooted in lessons from past failures, must drive collaborative translational efforts.

### 5.1 Limitations of BiTEs and CAR-T therapies

While BiTEs and CAR-T therapies targeting DLL3 have shown remarkable clinical promise, their limitations warrant careful scrutiny. For BiTEs, systemic toxicity remains a critical challenge—the trial reported cytokine release syndrome (CRS) in 52% of tarlatamab-treated patients (grade ≥3 in 1%) and Any-grade treatment-related adverse events in 90.7% (76). These adverse events stem from on-target, off-tissue activity due to low-level DLL3 expression in normal tissues (e.g., CNS, pancreas) and hyperactivation of circulating T cells (37). While BiTEs like DLL3-targeted constructs enhance T-cell activation, preclinical models suggest that SCLC tumors may compensate via alternative immunosuppressive pathways. In Chen et al. (2020), combining DLL3 BiTE (SC16.56) with PD-1 blockade improved antitumor efficacy compared to monotherapy *in vivo*, implying that PD-L1/PD-1 signaling remains a resistance mechanism even during BiTE treatment (109). Although the study did not directly measure PD-L1 levels post-BiTE, the synergy highlights the need for combinatorial approaches to counteract adaptive immune evasion.

CAR-T therapies face additional hurdles: antigen heterogeneity (only 60–70% of SCLC tumors exhibit homogeneous DLL3 expression) and T-cell exhaustion within the TME. Preclinical studies observed that DLL3-targeted CAR-T cells require repeated dosing to sustain efficacy in SCLC xenografts (41). Recent studies by Shirasawa et al. further reveal that spatial heterogeneity in DLL3 expression correlates with clonal evolution under therapeutic pressure. Their work demonstrates that DLL3High tumors, despite harboring higher neoantigen loads, develop resistance to immunochemotherapy through suppressed antigen-presenting functions (e.g., dendritic cell differentiation) and diminished T-cell infiltration. This paradox highlights the need for dynamic monitoring of DLL3 expression patterns to address evolving resistance (56). Strategies to overcome TME-mediated CAR-T exhaustion are being explored. For example, Jaspers et al. (2023) demonstrated that IL-18-secreting DLL3 CAR-T cells enhance T-cell activation and reduce exhaustion in SCLC preclinical models (99). Furthermore, metabolic engineering methods and epigenetic modifications, including enhancing mitochondrial function and altering the function of epigenetic enzymes, have demonstrated efficacy in preclinical studies but necessitate additional clinical validation (126, 127).

### 5.2 Clinical failure of Rova-T: beyond preclinical promise

The failure of Rova-T despite robust preclinical activity (80% tumor regression in PDX models) highlights translational challenges in ADC development. Key factors include (1) Target Heterogeneity: Only 68% patients had high DLL3 expression (defined as ≥75% of tumor cells) (52), but the phase III TAHOE trial enrolled patients regardless of DLL3 levels (70), diluting efficacy. Furthermore, DLL3 expression levels may demonstrate a downward trend following chemotherapy, thereby reducing the treatment window. (2) Payload Toxicity: The pyrrolobenzodiazepine (PBD) dimer caused dose-limiting vascular leak syndrome and thrombocytopenia, restricting therapeutic indices (33). In contrast, next-gen ADCs like FZ-AD005 employ topoisomerase inhibitors with narrower toxicity profiles (71). (3) Tumor Plasticity: Preclinical models overrepresented ASCL1-driven SCLC subtypes with stable DLL3 expression, whereas NEUROD1-dominant subtypes show epigenetic DLL3 suppression under therapeutic pressure (26). Emerging evidence suggests that chemotherapy-induced DLL3 downregulation may further contribute to Rova-T resistance. As highlighted by Su et al., platinum/etoposide regimens epigenetically suppress DLL3 expression via ASCL1 inhibition in NEUROD1-dominant SCLC subtypes (13, 57). This mirrors preclinical findings where chemotherapy reduced DLL3 membrane localization in SCLC PDX models, impairing Rova-T binding (33). These dynamics underscore the need for longitudinal DLL3 monitoring, as stable DLL3 expression was observed in <30% of post-chemotherapy SCLC biopsies in TRINITY. This plasticity underscores the need for real-time biomarker monitoring.

### 5.3 DLL3’s dual role: therapeutic paradox or contextual nuance?

DLL3’s conflicting roles—promoting metastasis via EMT yet correlating with immune evasion—reflect contextual signaling crosstalk. Mechanistically, DLL3 inhibits NOTCH-mediated differentiation in SCLC stem cells (95), fostering aggressiveness, while its high expression correlates with STAT3 activation and PD-L1 upregulation (56). In addition, Shirasawa et al. directly links DLL3High status to STAT3-driven PD-L1 amplification and suppressed antigen presentation (e.g., downregulated MHC-I/II and dendritic cell dysfunction), mechanistically explaining the ‘cold’ TME resistant to ICIs. Their transcriptomic data reveal that even neoantigen-rich DLL3High tumors evade immune recognition via STAT3/PD-L1 axis activation, reinforcing the need for combination strategies targeting both DLL3 and PD-L1 (56). This duality creates a therapeutic paradox: DLL3-targeted therapies may inadvertently select for PD-L1-high, immune-resistant clones. However, the relationship between molecular subtypes and PD-L1 expression remains controversial. In the Lang et al. study of surgically resected SCLC, neither tumoral nor stromal PD-L1 expression correlated with ASCL1, NEUROD1, or POU2F3 subtypes (128). Emerging data suggest that the interplay between DLL3 and PD-L1 may depend on tumor microenvironmental cues rather than intrinsic subtype biology. For instance, ASCL1-high tumors often exhibit neuroendocrine-driven immune exclusion, while POU2F3-driven tumors may lack PD-L1 due to reduced T-cell infiltration (110). These findings highlight the need for multimodal biomarker strategies integrating molecular subtyping and immune contexture analysis.

### 5.4 Emerging research trends and future directions

DLL3, expressed in most SCLC patients, is a critical therapeutic target driven by the transcription factor ASCL1, enhancing tumorigenic, clonogenic, and metastatic capacities in preclinical models (27, 53, 108, 129, 130). While its role in promoting SCLC progression via epithelial-mesenchymal transition (EMT) regulation has been proposed (27), clinical studies reveal no correlation between DLL3/ASCL1 expression and survival outcomes in limited- or extensive-stage SCLC (27, 56, 131). Intriguingly, molecular subtyping of SCLC tumors demonstrates DLL3’s absence in ASCL1/NEUROD1 double-negative subtypes but high expression in ASCL1- or NEUROD1-dominant tumors (110, 111), underscoring its complex diagnostic utility. Like PD-1 inhibitors, DLL3-targeted therapies face intrinsic resistance driven by MHC-I loss and terminally exhausted T-cell states. Preclinical data suggest that DLL3High/PD-L1High tumors amplify these mechanisms via STAT3/β-catenin crosstalk, necessitating combinatorial strategies such as DLL3 BiTEs with epigenetic modulators (e.g., HDAC inhibitors) or metabolic reprogramming of CAR-T cells (132). Recent advances in off-the-shelf iPSC-derived NK cell platforms offer a scalable solution to the manufacturing bottlenecks of autologous CAR-T/NK therapies (133, 134). Engineered iPSC-NK cells with stable DLL3-targeting CARs and enhanced metabolic fitness (e.g., IL-15/21 priming) demonstrate potent cytotoxicity against heterogeneous DLL3High/PD-L1High SCLC models while evading fratricide and exhaustion. These ‘universal’ cells could synergize with BiTEs to overcome TME immunosuppression, representing a paradigm shift in scalable DLL3-targeted immunotherapy. Future research must prioritize large-scale studies and standardized assays to clarify DLL3’s dual role as a therapeutic target and prognostic marker.

SCLC therapy development faces challenges including rapid disease progression, limited biomarker characterization, and treatment resistance (135–137). Although chemo-immunotherapy (e.g., atezolizumab with carboplatin/etoposide) has improved survival in ES-SCLC (18, 19, 138–141), identifying biomarkers to predict therapeutic response remains urgent. The divergent clinical trajectories of DLL3-targeted modalities—spanning ADCs, BiTEs, and CAR-based platforms—reflect both their unique therapeutic potentials and shared challenges. **Table 3** provides a critical synthesis of these approaches, contrasting their mechanisms, clinical performance, and translational bottlenecks. While ADCs like FZ-AD005 prioritize payload precision, BiTEs such as tarlatamab emphasize rapid immune engagement, and CAR-T/NK therapies aim for durable remissions. However, common barriers—including antigen heterogeneity, immunosuppressive TME, and on-target/off-tumor toxicity—demand innovative solutions. Future efforts must focus on biomarker-guided combinatorial strategies, such as pairing BiTEs with ICIs to amplify T-cell infiltration or engineering CAR-T cells with metabolic resilience to counteract TME-driven exhaustion. For instance, BI764532 enhances T-cell infiltration in SCLC tumors while upregulating PD-1/PD-L1, suggesting combinatorial potential with checkpoint blockade (37, 109). Similarly, combining DLL3 BiTEs with PD-1 inhibitors improves efficacy in xenograft models (85, 109), laying groundwork for clinical trials.

**Table 3:** Comparative analysis of DLL3-Targeted therapeutic modalities in SCLC.

Category	Agents	Mechanism	Key Efficacy	Major Toxicities	Clinical Stage	Advantages	Limitations
ADCs	Rova-T	Anti-DLL3 mAb + DNA crosslinker (PBD)	ORR: 14.3%, OS: 5.7 months, and PFS: 3.8 months in DLL3-high (TRINITY phase II) (29)	Grade 3–5 AEs: 63% (fatigue, photosensitivity reaction, and pleural effusion) (29)	Discontinued	Potent tumor-specific cytotoxicity; targets cancer stem cells (68)	Payload-related toxicity; uncontrollable adverse reactions (30)
	FZ-AD005	Fc-silenced anti-DLL3 mAb + topoisomerase I inhibitor (DXd; DAR=8.0)	Preclinical: potent tumor regression in PDX models (71)	No fatal cases or life-threatening toxicities (71)	Phase I (ongoing)	Improved safety profile; optimized DAR (71)	Limited clinical data; long-term safety unknown
BiTEs	Tarlatamab (AMG757)	DLL3/CD3 bispecific IgG with extended half-life	OS: 4.9 months (10mg),3.9(100mg) (phase II) (77)	CRS (manageable); neutropenia (76, 77)	FDA-approved	Rapid T-cell engagement; MHC-I independent (77)	Short serum half-life requires biweekly dosing
	BI764532	DLL3/CD3 IgG-like T-cell engager	Preclinical: Complete regression in xenografts (37)	Pending phase I data (79)	Phase I	Enhanced tumor penetration; preclinical synergy with ICIs (37)	Potential hematologic toxicity (preclinical) (78)
	HPN328	Trispecific TCE (DLL3/CD3/albumin-binding)	Target lesion reduction: 40% in SCLC (phase I/II interim) (84)	No grade ≥3 AEs in responders (84)	Phase I/II	Extended half-life; compact molecular design (83)	Limited efficacy data in NECs
CAR-T/NK	AMG119	DLL3-targeted CAR-T with 4-1BB costimulation	Preclinical: Durable tumor regression in xenografts (97)	No dose-limiting toxicities or grade 4/5 AEs (97)	Discontinued	Better T cell persistence (97)	Manufacturing complexity; T-cell exhaustion in TME (40)
	IL-18-engineered CAR-T	DLL3 CAR-T + IL-18 secretion	Preclinical: Enhanced T-cell activation; synergy with anti-PD-1 (99)	N/A (preclinical)	Preclinical	Reverses TME immunosuppression; reduces exhaustion (99)	Unclear clinical translatability
	CAR NK-92 cells	Allogeneic NK cells engineered with DLL3-specific CAR + NKG2D/2B4 costimulatory domains	Preclinical: Potent cytotoxicity in systemic SCLC models (39)	Minimal CRS risk (preclinical) (39)	Phase I (NCT05507593)	Off-the-shelf availability; dual killing mechanisms (101)	Limited infiltration in fibrotic tumors (39)

AE, adverse event; ORR, Objective Response Rate; OS, Overall Survival; CRS, Cytokine Release Syndrome; DAR, Drug-to-Antibody Ratio; PDX, Patient-Derived Xenograft; NEC, Neuroendocrine Carcinoma.

**Figure 2:**
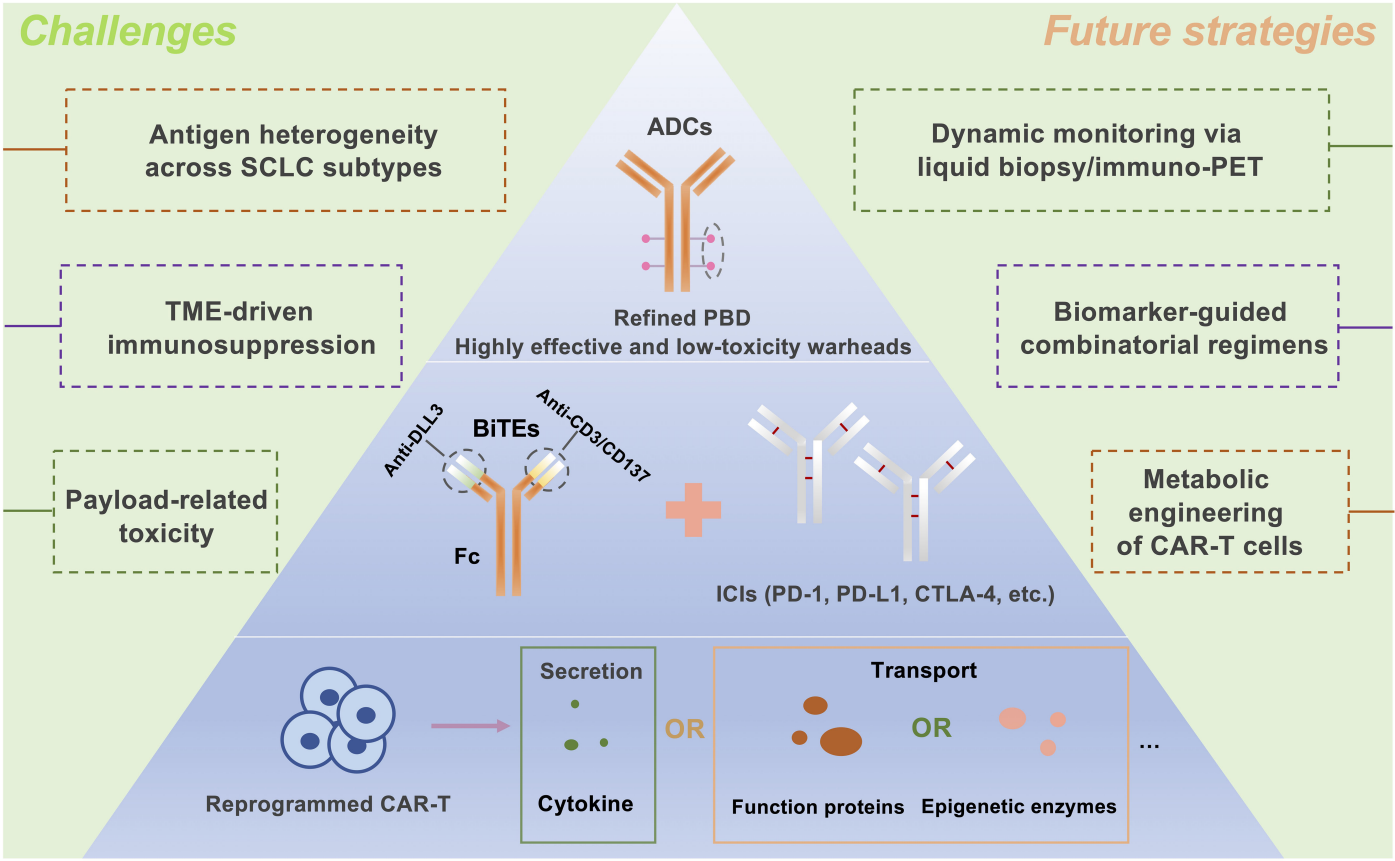
Future Directions in DLL3-Targeted Immunotherapy for Small Cell Lung Cancer: Integrated Strategies to Overcome Therapeutic Challenges. The future development of DLL3-targeted immunotherapies for small-cell lung cancer (SCLC) will focus on three key strategies: Antibody-Drug Conjugates (ADCs): Designing ADCs with potent yet minimally toxic payloads, such as pyrrolobenzodiazepine (PBD), to selectively target DLL3-expressing tumors. BiTE and Immune Checkpoint Inhibitor (ICI) Combination: Enhancing antitumor efficacy by combining BiTEs with ICIs, targeting PD-1, PD-L1, or CTLA-4 to boost immune response. Metabolically Reprogrammed CAR T Cells: Engineering chimeric antigen receptor (CAR) T cells to enhance their persistence and functionality in the tumor microenvironment, potentially through cytokine secretion or epigenetic metabolic modifications. ADCs, Antibody-drug conjugates; BiTEs, Bispecific T-cell engagers; CAR T, Chimeric antigen receptor T-cell; PBD, Pyrrolobenzodiazepine; ICIs, Immune checkpoint inhibitors; PD-1, Programmed cell death protein 1; PD-L1, Programmed cell death ligand 1; CTLA-4, Cytotoxic T lymphocyte-associated protein 4.

CAR T-cell therapy, distinct from BiTEs in its costimulation-dependent T-cell activation (109), faces hurdles in solid tumors due to antigen heterogeneity and immunosuppressive microenvironments (41, 92, 142–145). DLL3’s tumor-specific expression in SCLC offers a viable target, while engineering strategies such as cytokine-secreting “armored” CARs (e.g., IL-12 or IL-18 producers) show promise in overcoming TME resistance (96, 146–149). Preclinical success with IL-18-expressing DLL3 CAR T cells in SCLC (99) suggests similar potential for IL-12 variants. Future directions include developing multifunctional DLL3-targeted CAR T cells incorporating metabolic reprogramming, epigenetic modifiers, or dual cytokine secretion to enhance potency and durability (**Figure 2**).

## Glossary

ADC: antibody-drug conjugate
ADCC: antibody-dependent cell-mediated cytotoxicity
ASCL1: achaete-scute homolog 1
ADCP: antibody-dependent cell phagocytosis
BiTE: bispecific T-cell engager
CAIX: carbonic anhydrase IX
CAR T: chimeric antigen receptor T
CDX: cell line-derived xenograft
CE: carboplatin and etoposide
CHK1: checkpoint kinase 1
CRPC: castration-resistant prostate cancer
CTCs: circulating tumor cells
CTLA-4: cytotoxic T lymphocyte associate protein-4
DLL3: delta-like canonical notch ligand 3
DAR: drug antibody ratio
DFO: desferrioxamine
DFS: disease-free survival
DXd: deruxtecan
EMT: epithelial-mesenchymal transition
ES-SCLC: extensive-stage SCLC
Fc: fragment crystallization
GEP-NENs: gastroenteropancreatic neuroendocrine neoplasms
HCC: hepatocellular carcinoma
HER2: human epidermal growth factor receptor 2
H3K27me3: histone H3 lysine 27 methylation
IBC: invasive breast cancer
ICIs: immune checkpoint inhibitors
IDH: isocitrate dehydrogenase
IHC: immunohistochemistry
LCNEC: large cell neuroendocrine carcinoma
LS-SCLC: limited-stage SCLC
mAb: monoclonal antibody
MHC: major histocompatibility complex
MTC: medullary thyroid carcinoma
NECs: neuroendocrine carcinomas
NEPC: neuroendocrine prostate cancer
NIR-PIT: near-infrared photoimmunotherapy
NKG2D: natural-killer group 2, member D
NSCLC: non-small cell lung cancer
OS: overall survival
PARP1: poly (ADP-ribose) polymerase-1
PBD: pyrrolobenzodiazepine.
PD-1: programmed cell death protein 1
PD-L1: programmed cell death ligand 1
PDX: patient-derived xenograft
PET: positron emission tomography
Rova-T: rovalpituzumab tesirine
SCC: small-cell carcinomas
scFv: single-chain fragment variable
SCLC: small cell lung cancer
SPs: surface proteins
TCE: T-cell engager
TME: tumor microenvironment
TriTAC: tri-specific T-cell activation construct
TRUCKs: T cell redirection for universal cytokine-mediated killing
